# *nosX* is essential for whole-cell N_2_O reduction in *Paracoccus denitrificans* but not for assembly of copper centres of nitrous oxide reductase

**DOI:** 10.1099/mic.0.000955

**Published:** 2020-09-04

**Authors:** Sophie P. Bennett, Maria J. Torres, Manuel J. Soriano-Laguna, David J. Richardson, Andrew J. Gates, Nick E. Le Brun

**Affiliations:** ^1^​ Centre for Molecular and Structural Biochemistry, School of Chemistry, University of East Anglia, Norwich Research Park, Norwich, NR4 7TJ, UK; ^2^​ Centre for Molecular and Structural Biochemistry, School of Biological Sciences, University of East Anglia, Norwich Research Park, Norwich, NR4 7TJ, UK

**Keywords:** copper, denitrification, nitrous oxide, nitrous oxide reductase

## Abstract

Nitrous oxide (N_2_O) is a potent greenhouse gas that is produced naturally as an intermediate during the process of denitrification carried out by some soil bacteria. It is consumed by nitrous oxide reductase (N_2_OR), the terminal enzyme of the denitrification pathway, which catalyses a reduction reaction to generate dinitrogen. N_2_OR contains two important copper cofactors (Cu_A_ and Cu_Z_ centres) that are essential for activity, and in copper-limited environments, N_2_OR fails to function, contributing to rising levels of atmospheric N_2_O and a major environmental challenge. Here we report studies of *nosX*, one of eight genes in the *nos* cluster of the soil dwelling α-proteobaterium *Paraccocus denitrificans*. A *P. denitrificans* Δ*nosX* deletion mutant failed to reduce N_2_O under both copper-sufficient and copper-limited conditions, demonstrating that NosX plays an essential role in N_2_OR activity. N_2_OR isolated from *nosX*-deficient cells was found to be unaffected in terms of the assembly of its copper cofactors, and to be active in *in vitro* assays, indicating that NosX is not required for the maturation of the enzyme; in particular, it plays no part in the assembly of either of the Cu_A_ and Cu_Z_ centres. Furthermore, quantitative Reverse Transcription PCR (qRT-PCR) studies showed that NosX does not significantly affect the expression of the N_2_OR-encoding *nosZ* gene. NosX is a homologue of the FAD-binding protein ApbE from *Pseudomonas stutzeri *, which functions in the flavinylation of another N_2_OR accessory protein, NosR. Thus, it is likely that NosX is a system-specific maturation factor of NosR, and so is indirectly involved in maintaining the reaction cycle of N_2_OR and cellular N_2_O reduction.

## Introduction

Nitrous oxide is a potent greenhouse gas which has rapidly increased in the atmosphere over the past century [1]. The rise in N_2_O coincides with the introduction and application of anthropogenic nitrogen species in agriculture, to improve crop yield and ultimately feed the growing global population [2, 3]. Of the total N_2_O released, 40 % is produced by soil bacteria [4]. Soil dwelling denitrifying micro-organisms such as *Paracoccus denitrificans* consume nitrate as an alternative electron acceptor during anaerobic growth conditions. N_2_O is an intermediate substrate in the denitrification pathway; it is reduced to N_2_ by the copper enzyme nitrous oxide reductase (N_2_OR). N_2_OR-containing bacteria can be separated into two clades, and a feature that distinguishes the clades is the ability of the micro-organism to produce and consume, or only consume, N_2_O [5, 6]. Clade-I members are complete denitrifiers with the nitrite reductase genes *nirS* or *nirK* present in their genome. In contrast, about half of the clade-II members are non-denitrifying N_2_O reducers, and are therefore N_2_O sinks [5]. Ammonia-oxidizing bacteria (AOB) are another microbial source of N_2_O in coastal ecosystems, through a process named ‘nitrifier denitrification’. However, they do not harbour genes encoding N_2_O reduction activity [7]. Environmental factors such as soil pH, Cu content, and moisture impact on N_2_O emissions from soil [8–10]. In order to identify N_2_O mitigation strategies, we are trying to understand the optimal genetic components needed to biologically remove N_2_O.

Nitrous oxide reductase (N_2_OR) is a homo-dimeric, ~120 kDa, multi-Cu protein. Each monomer contains two Cu cofactors, the Cu_A_ and Cu_Z_ centres, responsible for electron transfer and the catalytic reduction of N_2_O, respectively. The Cu_A_ centre is a *bis*-thiolate-bridged di-nuclear Cu centre, accommodated within a cupredoxin fold domain, similar to that of subunit II of cytochrome *c* oxidase. The Cu_Z_ centre is a unique [Cu-S] cluster ligated by seven conserved histidine residues within a β-barrel domain. It comprises four Cu atoms and one or two sulphur atoms, depending on the purification method [11–13]. Notably, the subunits of the active homodimer are orientated in a head to tail configuration, with one Cu_A_ centre in close proximity to the Cu_Z_ centre of the other monomer. N_2_OR is encoded by the *nosZ* gene, which, in denitrifying organisms such as *Paracoccus denitrificans* and *Pseudomonus stutzeri*, is translated and exported through the twin-arginine transport [14] pathway to the periplasm, as a folded apo-protein, before acquiring its Cu cofactors. Consistent with this, a TAT signal leader sequence mutant accumulated unprocessed, dimeric, apo-protein in the cytoplasm of the cell [15]. In contrast, the N_2_OR of clade-II members are transported through the Sec pathway [16]. The functional significance of this is currently unknown.

The *nosZ* gene is found among the *nos* gene cluster (NGC), which comprises eight genes in *P. denitrificans: nosCRZDFYLX*. The *nosC* and *nosR* genes are copper responsive in *P. denitrificans* and function in the regulation of *nosZ* transcription. During Cu limitation, *nosCR* transcription is increased, whilst *nosZ* transcription is reduced [17]. In *Pseudomonas stutzeri *, NosR is a cytoplasmic membrane protein with two soluble domains located at either side of the membrane: the N-terminal periplasmic domain covalently binds a flavin mononucleotide, while the C-terminal cytoplasmic domain binds two [4Fe-4S] clusters [18]. The *P. denitrificans* homologue (44.3 % identical) is predicted to have similar features. The function of NosR is not well understood; in addition to the regulatory role mentioned above, it is important for whole-cell N_2_O reduction [17, 18], with evidence indicating that it is not involved in the assembly of the Cu centres of N_2_OR, but may be the physiological electron donor to NosZ [18].

*nosDFY* encode a cytoplasmic membrane spanning ABC-type transporter that functions in the maturation of the Cu_Z_ centre of N_2_OR, as illustrated by an insertional mutation in *P. stutzeri nosD,* which produced an N_2_OR without the key spectroscopic signal of the Cu_Z_ centre [19]. Similarity to mitochondrial ABC transporters that export a sulphur species to the cytoplasm for iron-sulphur cluster biogenesis suggests a role for NosDFY in providing the essential sulphur atoms of the catalytic Cu_Z_ centre [20]. The *nosL* gene is well conserved across NGCs and is essential for whole-cell N_2_O reduction in *P. denitrificans *. NosL is a Cu-binding lipoprotein, putatively anchored to the outer membrane of the cell. The properties of N_2_OR purified from a PdΔ*nosL* strain revealed that Cu-binding NosL is a component of the Cu_Z_ maturation apparatus under Cu replete conditions and, more importantly, is an essential maturation factor for both Cu centres during Cu limitation [21].

**Fig. 1.**
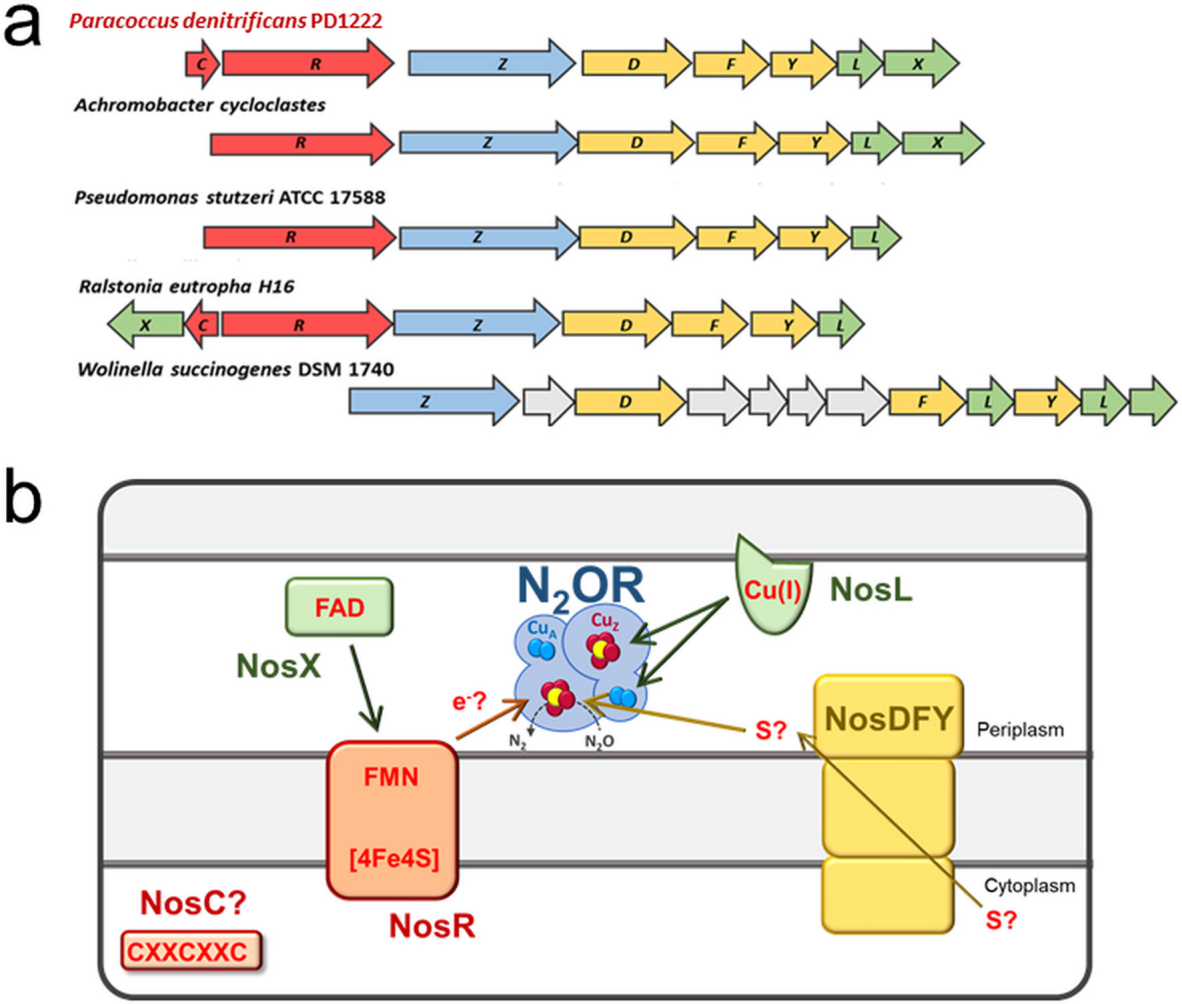
(a) Comparison of NGCs from clade-I nitrous-oxide-reducing bacteria (*P. denitrificans, Achromobacter cycloclastes*, *Pdeudomonas stutzeri*, *Ralstonia eutropha *) and the clade-II member *Wolinella succinogenes *. (b) The core *nosZDFYL* genes encode the nitrous oxide reductase polypeptide (NosZ), an ABC transporter complex (NosDFY) that is essential for Cu_Z_ centre maturation, and a Cu centre maturation factor (NosL). The *nosR* and *nosX* genes are less conserved across the two clades. NosR is a transmembrane iron-sulflur cluster containing protein with an FMN moiety, which is obtained from an ApbE-type flavinyltransferase (proposed as NosX here). Together the function of these proteins may involve supplying electrons to N_2_OR for catalytic turnover and, where absent in the NGC, a homologue is likely to be found elsewhere in the genome.

The *nosX* gene is predominantly found in α- and β-proteobacterial NGCs in clade I but does not feature among γ-proteobacteria nor clade-II NGCs (Fig. 1). NosX is a soluble protein of ~30 kDa, which is exported to the periplasm by the Tat pathway. Previously, it was reported that insertional mutagenesis of *P. denitrificans nosX* resulted in wild-type-like growth [22]. Interruption of both *nosX* and the homologue *nirX* did, however, present a Nos-negative (Nos^-^) phenotype, leading to the conclusion that NosX and NirX are functional homologues [22]. Furthermore, this study demonstrated that the *nosX nirX* double mutant strain contained N_2_OR that was deficient in its Cu_A_ centre, implicating these proteins in copper cofactor assembly [22].

The γ-proteobacterium *P. stutzeri *, which does not feature *nosX* in its NGC, instead contains a NosX homologue encoded elsewhere on the genome. The protein, called ApbE, was shown to be a FAD-binding flavinyl transferase that serves as a flavin donor to NosR, which in turn activates N_2_OR [23]. *Ps*ApbE and *Pd*NosX share 32 % amino acid homology, in particular the conservation of key amino acid residues associated with flavin binding suggest that their roles are similar while their genetic context implies they may differ in system specificity. Here, we present a re-examination of the role of NosX in *P. denitrificans *, through the analysis of full *nosX* deletion in *P. denitrificans,* in terms of cell growth and the properties of N_2_OR purified from an unmarked mutant background. The data show that NosX is essential for N_2_OR activity and cannot be substituted by NirX. Furthermore, NosX plays no role in assembly of the NosZ Cu cofactors, nor does it have a major function in the regulation of *nosZ* expression. Instead, the role of NosX is consistent with a system-specific maturation factor for NosR to support the activity of NosZ *in vivo*.

## Methods

### Construction and complementation of a *nosX*-deficient strain of *P. denitrificans*

A double allelic exchange method was employed to generate a whole *nosX* gene deletion strain (Table S1, available in the online version of this article), as described previously [17, 21]. Briefly, the suicide plasmid pK18*mobsacB* containing DNA regions that flank the *nosX* gene (pSPBN4) was conjugated into PD1222 using the *E. coli* helper plasmid pRK2013. Single cross-over recombination events resulted in Spec^R^/Km^R^ transconjugants, from which a double cross over mutant (Spec^R^), named PD2502, was generated. The mutated region was PCR amplified and confirmed by sequencing.

*Pd*Δ*nosX* (PD2502) was complemented *in trans* using pSPBN5, which contains the coding sequence of Pden_4214. The gene was synthesized by Genscript with flanking 5′ *Nde*I and 3′ *Eco*RI restriction sites and subcloned into a taurine inducible modified pLMB509 derivative with gentamycin resistance (20 µg ml^−1^) to generate pSPBN5. The complementation plasmid was conjugated into the mutant strain using the helper *E. coli* pRK2013 strain, with successful conjugants identified as Spec^R^/Gm^R^. Expression of *nosX* from the plasmid was induced by adding 1 mM taurine to the medium at the start of growth.

### Growth and phenotypic analysis of cultures

Anaerobic minimal media batch cultures (400 ml) were grown in sealed Duran flasks fitted with a septum seal to allow for gas-tight sample extraction. Minimal media consisted of: 30 mM succinate, 20 mM nitrate, 11 mM dihydrogen orthophosphate, 29 mM di-sodium orthophosphate, 0.4 mM magnesium sulphate, 1 mM ammonium chloride, pH 7.5. The minimal media was supplemented with a 2 ml l^−1^ Vishniac and Santer trace element solution [24] where copper sulphate was present (Cu-sufficient, 12.8 µM) or excluded (Cu-limited, <0.5 µM) from the original recipe. Media were inoculated using a 1 % inoculum from a starter culture to give a starting OD_600 nm_ of ~0.02 and incubated at 30 °C. Samples of the liquid culture were taken in 1 ml aliquots and OD_600 nm_ measured. The 3 ml gas samples were removed from the headspace of the cultures and stored in pre-evacuated 3 ml Exetainer vials. A 50 µl gas sample was injected into a Clarus 500 gas chromatograph (PerkinElmer) equipped with an Elite-PLOT Q (30 m×0.53 mm internal diameter) and an electron capture detector. Carrier gas was N_2_, make-up gas was 95 % (v/v) argon, 5 % (v/v) methane. Standards containing N_2_O at 0.4, 5, 100, 1000, 5000 and 10 000 p.p.m. (Scientific and Technical Gases) were measured and total N_2_O was determined as previously described [17].

### Purification and characterization of affinity-tagged N_2_OR from *P. denitrificans* strains

Plasmid pMSL002, which encodes NosZ (N_2_OR) with a C-terminal Strep*-*tag II, was conjugated into wild-type (PD1222), *Pd*Δ*nosZ* (PD2303) and *Pd*Δ*nosX* (PD2502) strains using the *E. coli* pRK2013 helper strain. Strep-tagged N_2_OR was overproduced and purified as previously described [21]. Briefly, this involved applying the soluble portion of cell lysates to a Hi-Trap HP Strep II affinity column (5 ml, GE Healthcare) and eluting with 20 mM HEPES, 150 mM NaCl and 2.5 mM desthiobiotin, pH 7.2, before exchanging into 20 mM HEPES, 150 mM NaCl, pH 7.2. Sample purity was confirmed using SDS-PAGE analysis and LC-MS. Protein concentrations were determined using the Bradford assay (BioRad) [25] and bovine serum albumin as a protein standard.

UV-visible absorbance spectra of N_2_OR-Strep-tag II from different backgrounds were recorded on a Jasco V-550 spectrophotometer. Samples were made anaerobic by sparging with nitrogen gas for 5 min and oxidized or reduced with 5 mg ml^−1^ stocks of potassium ferricyanide and sodium dithionite, respectively, in 20 mM HEPES, 150 mM NaCl, pH 7.5, by titrating concentration equivalents. Total copper content of the protein was determined using a colorimetric bathocuproinedisulfonic acid (BCS) assay as previously described [21].

Activities of N_2_OR-Strep-tag II isolated from different backgrounds were determined using an adapted methyl viologen assay [26, 27] in which samples were pre-incubated with a 500-fold excess of reduced methyl viologen for 150 min. Reaction was initiated by adding N_2_O saturated buffer and the oxidation of blue (reduced) methyl viologen to its oxidized colourless form was followed at 600 nm as a function of time and data converted to specific activity using ε_600 nm_=13 600 M^−1^ cm^−1^ for the reduced methyl viologen cation radical [27].

### RNA isolation, cDNA synthesis and qRT-PCR experiments

Expression of the *nosZ* gene was determined by qRT-PCR, using an AriaMx Real-Time PCR System G9930A (Agilent Technologies). The *nosX* mutant and PD1222 wild-type strains were cultivated under anoxic conditions as mentioned above for 12 h, reaching final OD_600 nm_ of 0.6. Total RNA extraction, RNA quality and integrity assays, and RNA quantification were performed using the methodology previously described [17]. Briefly, 2 µg of total RNA were used for cDNA synthesis using RevertAid First Strand cDNA synthesis kit (Thermo Scientific) and random hexamers following the supplier’s instructions. qRT-PCR reactions were run in triplicate in a total volume of 20 µl containing 10 µl of SensiFAST SYBR No-ROX Mix (Bioline), 0.7, 7 or 70 ng of cDNA and 2 µM of each primer. Melting curves were generated to verify the specificity of each amplification reaction. Expression of *nosZ* gene was determined using the oligonucleotide pair nosZ2F/nosZ2R [17] and normalized against the housekeeping gene *gapA* (glyceraldehyde-3-phosphate dehydrogenase; GAPDH1F/GAPDH1R [17]). The changes in gene expression were analysed accordingly to Plaffl methodology [28]. The data presented correspond to the average of three independent biological replicates.

## Results

### NosX is essential for whole-cell N_2_O reduction in *P. denitrificans*

**Fig. 2.**
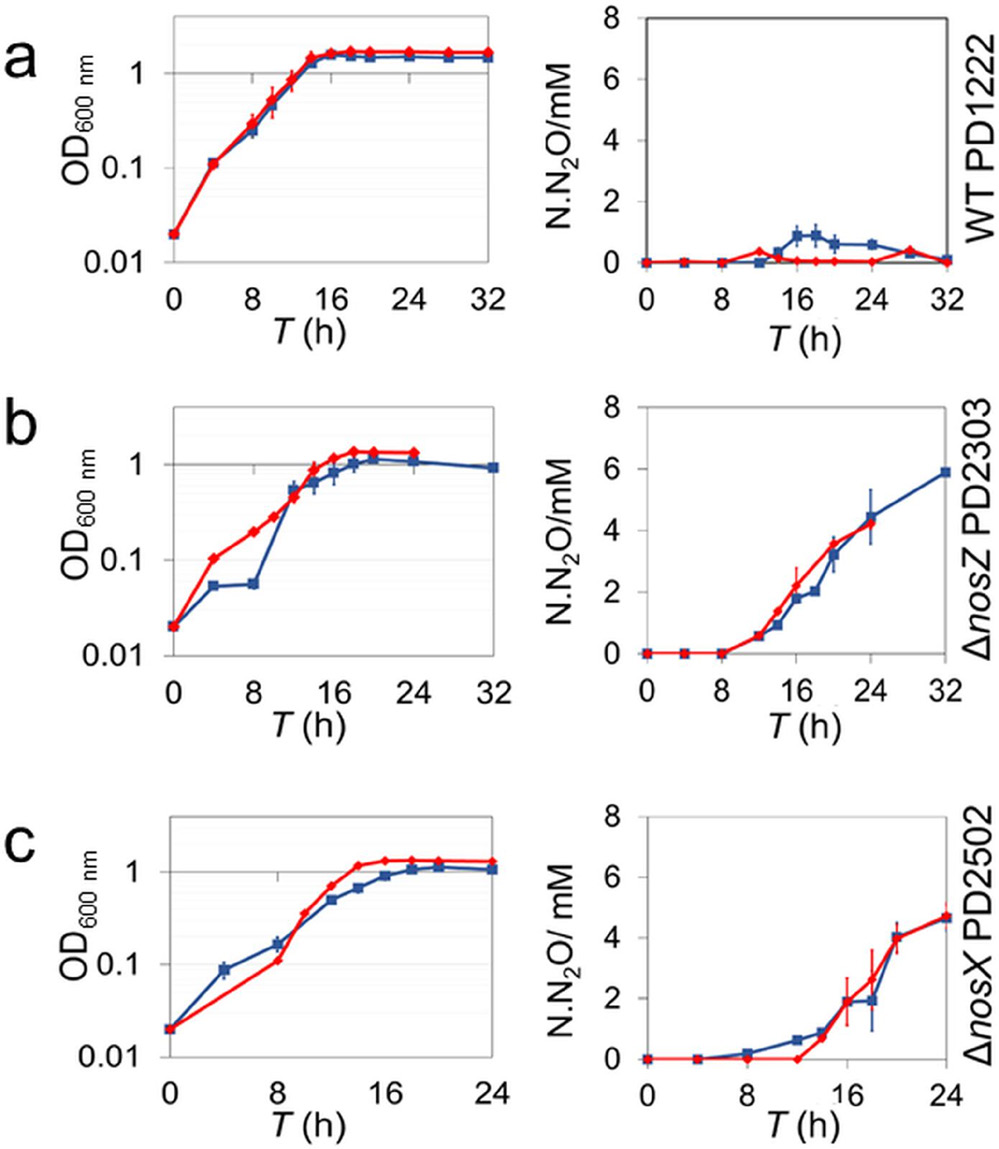
Growth and N_2_O production characteristics of *P. denitrificans* strains. (a) OD_600 nm_ as a function of time (left) and N_2_O emissions as N.N_2_O (millimolar N in the form of N_2_O, right) for wild-type PD1222 grown in anaerobic batch culture in Cu-sufficient media and Cu-limited media. (b) and (c) As in (a) but for Δ*nosZ* deletion mutant PD2303, and Δ*nosX* deletion mutant PD2502, respectively. Cultures were grown in triplicate and bars represent se.

Wild-type *P. denitificans* (PD1222), Δ*nosZ* (PD2303, missing the gene encoding N_2_OR) and Δ*nosX* (PD2502, missing the gene Pden_4214) were grown in batch culture, in minimal medium, under Cu-sufficient and limited conditions. The wild-type culture produced a small amount of N_2_O (~1 mM) in Cu-deficient conditions, but this was no longer detected as the culture moved into the stationary phase of growth Fig. 2. A N_2_OR-negative phenotype (Nos^-^), in terms of growth and N_2_O production, was observed in the Δ*nosZ* strain under both Cu regimes. For the Δ*nosX* strain, growth was affected both under Cu-sufficient and limited conditions, and N_2_O levels were similar to those of the Δ*nosZ* strain, demonstrating the absence of a functioning enzyme.

**Fig. 3.**
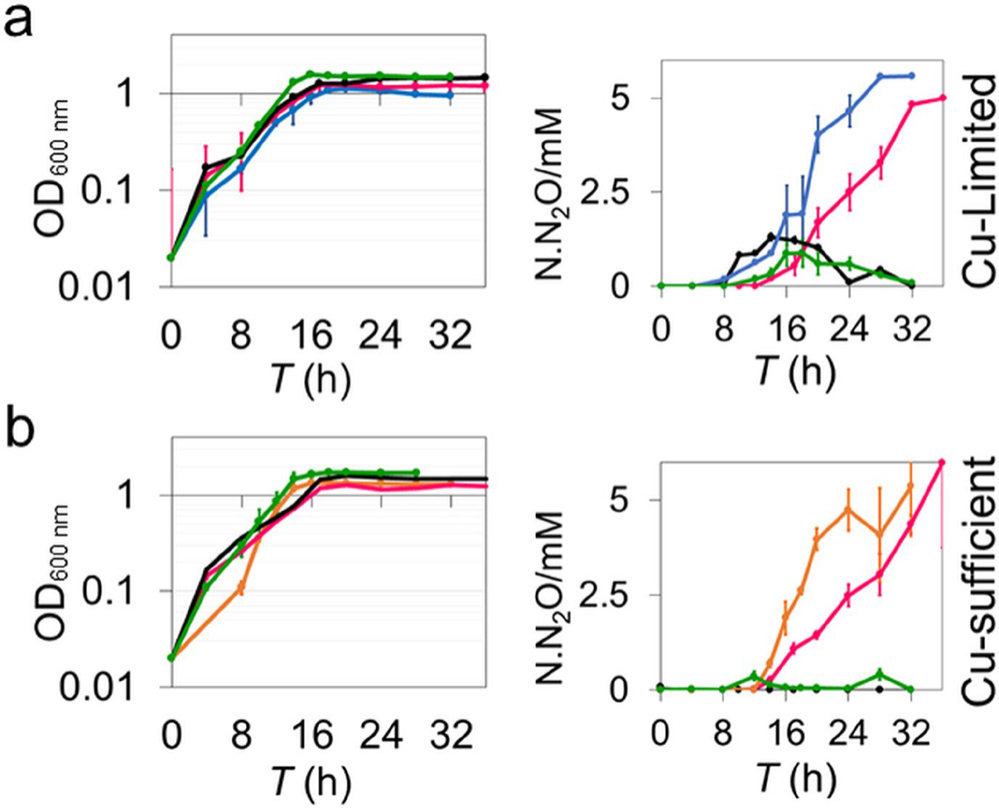
Complementation of the *nosX* mutant. (a) Growth characteristics (optical density, OD_600 nm_), left, and N_2_O production (N.N_2_O, mM N in the form of N_2_O), right for the mutant Δ*nosX* PD2502 complemented under (a) Cu-limited, and (b) Cu-sufficient conditions in anaerobic batch culture. The pSPBN5 plasmid was conjugated into the Δ*nosX* PD2502 strain and cultured in the absence of taurine and in the presence of 1 mM taurine. For reference, the Δ*nosZ* PD2303 strain and wild-type PD1222 are shown. Experiments were repeated in triplicate and bars represent se.

The Nos^-^ phenotype of the Δ*nosX* strain was almost fully complemented under both Cu regimes by a plasmid-borne *nosX* gene copy (pSPBN5) expressed *in trans* from a taurine inducible promoter (Fig. 3), demonstrating that the Nos^-^ phenotype is associated with the absence of *nosX* and not a downstream effect of the deletion. The data demonstrate that the *nosX* deletion mutant strain of *P. denitrificans* is unable to catalyse N_2_O reduction. This is in contrast to a previous study by Saunders and co-workers [22] involving a marked *nosX* deletion, where it was concluded that that NosX and NirX are functionally redundant, such that only one is required for N_2_O reduction.

### NosX is not involved in maturation of either Cu cofactor in N_2_OR

Three possible explanations for the Nos^-^ phenotype in the Δ*nosX* mutant are apparent: the incomplete maturation/assembly of copper centres of N_2_OR; the failure to activate N_2_OR catalytic activity, for example through disruption of supply of electrons; or, the severe down-regulation of *nosZ* transcription. To investigate this further, a C-terminal strep II-tagged N_2_OR was purified from the Δ*nosX* mutant strain and the properties of the N_2_OR analysed with respect to the status of the Cu_A_ and Cu_Z_ centres.

**Fig. 4.**
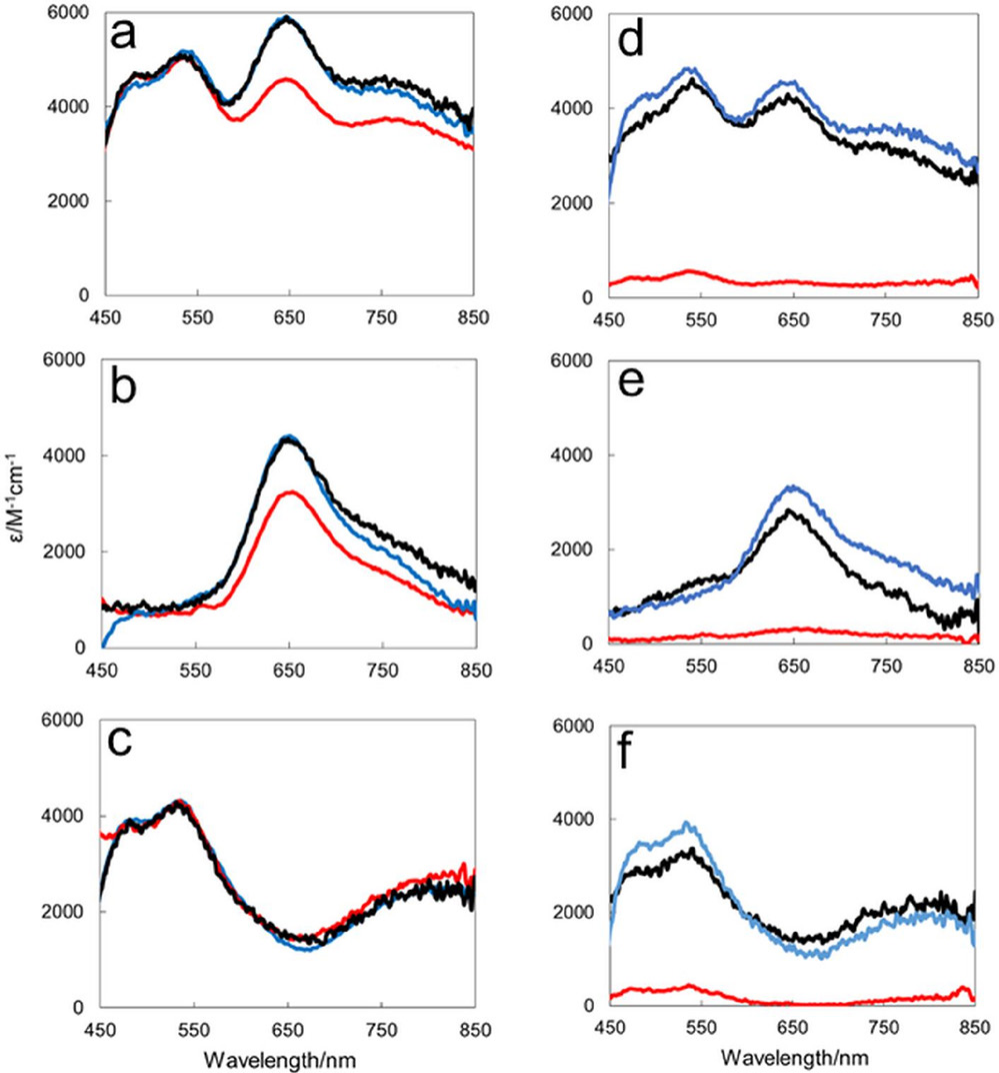
UV-visible absorbance characterisation of strep-tagged N_2_OR purified from different *P. denitrificans* backgrounds. Data are shown for N_2_OR from wild-type PD1222, Δ*nosX* PD2502 and Δ*nosZ* PD2303 in 20 mM HEPES, 150 mM NaCl, pH 7.2. Spectra of ferricyanide-oxidized (a), sodium dithionite-reduced (b) and the oxidized minus reduced difference (c) are shown for enzymes isolated from cultures grown under Cu-sufficient conditions. Equivalent spectra (d–f), respectively, were measured for enzymes isolated from cultures grown under Cu-limited conditions.

Aerobically purified N_2_OR, also known as the pink form of N_2_OR, has been spectroscopically well characterized and all oxidized spectra were normalised to ε_580 nm_5000 M^−1^ cm^−1^ per monomer, as described by Rasmussen *et al*. [13]. Absorbance spectra of N_2_OR enzymes isolated from cultures grown under Cu-sufficient conditions are shown in Fig. 4a. Spectra of N_2_OR from wild-type cells and Δ*nosX* and Δ*nosZ* mutants have features at 480, 540 and 640 nm, in agreement with the previous literature on N_2_OR from *P. denitrificans* [21], *P. pantotrophus* (*Pp*N_2_OR) [13], *Pseudomonas stutzeri* (*Ps*N_2_OR) [29], *Pseudomonas nautica* (*Pn*N_2_OR) [30], *Achromobacter cycloclastes* (*Ac*N_2_OR) [31] and *Marinobacter hydrocarbonoclasticus* (*Mh*N_2_OR) [32]. Features in the absorption spectrum at these wavelengths arise from S^2-^ to Cu(II) charge-transfer bands and additional optical bands due to interactions between the Cu(I) and Cu(II) ions of the centres [13]. Spectra of N_2_OR isolated from wild-type cells have lower extinction coefficients than those from the mutant strains, suggesting that it contains lower levels of Cu cofactors.

The Cu content of all isolated N_2_OR enzymes was determined (Table 1), confirming that enzymes isolated from Δ*nosZ* and Δ*nosX* mutants are replete with Cu, while that from wild-type cells contains slightly lower amounts, consistent with the absorption spectra. N_2_OR activity was measured using a methyl viologen assay in which the reduced MV extinction coefficient, ε_600 nm_ = 13 600 M^−1^ cm^−1^ [27], was used to quantify activity, and N_2_OR was pre-incubated with a 500-fold excess reduced methyl viologen (MV) before initiating the reaction with N_2_O (Table 1). Each N_2_OR sample was active, with values for the enzyme from the wild-type and Δ*nosZ* strains consistent with those previously reported [21, 26, 30]. Activity for N_2_OR from the Δ*nosX* mutant was similar to that from wild-type, even though it contained significantly more Cu, suggesting that the enzyme from the Δ*nosX* mutant has a slightly lower activity.

**Table 1.** Summary of some characteristics of strep-tagged N_2_OR purified from *P. denitrificans* strains PD1222, PD2502 and PD2303

	Cu ions/monomer*		Specific activity† (µmol N_2_O min^−1^ mg ^−1^)
	Cu-sufficient	Cu-limited	
Wild-type PD1222/pMSL002 (StrepII tagged-NosZ)	5.6±0.1	0.4±0.27	171±13
Δ*nosX*/pMSL002	6.4±0.2	4.2±0.2	172±12
Δ*nosZ*/pMSL002	5.9±0.6	4.8±0.4	196±9

*Total copper per monomer was determined using the BCS Cu assay (see Methods).

†N_2_O reductase activity was determined for enzymes isolated from cultures grown under Cu-sufficient conditions using a reduced methyl viologen assay (µmol N_2_O min^−1^ mg^−1^ enzyme). Proteins were pre-incubated with a 500-fold excess reduced methyl viologen for 150 min prior to activity assay. All reactions were carried out in triplicate and sd is shown. nd. The data show that even though the Δ*nosX* strain has a Nos^-^ phenotype, N_2_OR isolated from it is fully or close to fully active in an *in vitro* assay.

Reduction of N_2_OR samples with dithionite leads to reduction of the Cu_A_ centre to a [Cu^1+^:Cu^1+^] diamagnetic species, which is colourless and thus does not contribute in the visible region of the absorbance spectrum. Thus, in Fig. 4b, bands at 480, 540 and 900 nm are lost to leave a Cu_Z_^*^ signature, consisting of a peak at 640 nm, in agreement with the literature for pink N_2_OR [13]. The oxidized minus reduced difference spectrum, Fig. 4c, revealed the spectrum due to the Cu_A_ centre. The close similarity of spectral form and absorption extinction coefficients for N_2_OR from Δ*nosZ* and Δ*nosX* mutants demonstrate that the assembly of the Cu cofactors of N_2_OR is not affected by the *nosX* deletion when grown under Cu sufficiency [13].

An equivalent spectroscopic analysis of N_2_OR enzymes isolated from cultures grown under Cu limitation (Fig. 4d–f) revealed spectra similar to those of Fig. 4a–c for enzymes from Δ*nosZ* and Δ*nosX* mutants, but with lower extinction coefficients, suggesting lower incorporation of Cu. Spectra for enzyme isolated from wild-type cultures, however, indicate very low levels of Cu incorporation. Determination of Cu content (Table 1) revealed that N_2_OR from Δ*nosZ* and Δ*nosX* mutants contain ~4 Cu per N_2_OR monomer, while that recovered from wild-type cells contains <1 Cu per monomer, consistent with absorbance data (Fig. 4d–f). As above, the close similarity between N_2_OR enzymes isolated from Δ*nosZ* and Δ*nosX* mutants demonstrate that NosX does not play a role in assembly of the Cu cofactors of N_2_OR under Cu-limited conditions.

### NosX has a minor effect on transcription of *nosZ* under Cu-sufficient conditions

The data presented in Fig. 4, Table 1 revealed some variability in the extent to which Cu_Z_ centres are assembled in enzymes isolated from different strains and grown under different conditions; specifically, plasmid-encoded strep-tagged N_2_OR isolated from wild-type cells contained fewer Cu_Z_ centres than that from the two mutants. Thus, the Δ*nosX* mutant behaves similarly to the Δ*nosZ* mutant, in which chromosomal *nosZ* is missing. This suggests that there may be fewer chromosomally encoded versions of N_2_OR in the *nosX* mutant than in wild-type cells, as would be expected if *nosZ* expression is perturbed in the *nosX* mutant. Less chromosomally encoded N_2_OR would provide less competition for Cu, leading to greater incorporation of Cu into the plasmid-encoded N_2_OR.

**Fig. 5.**
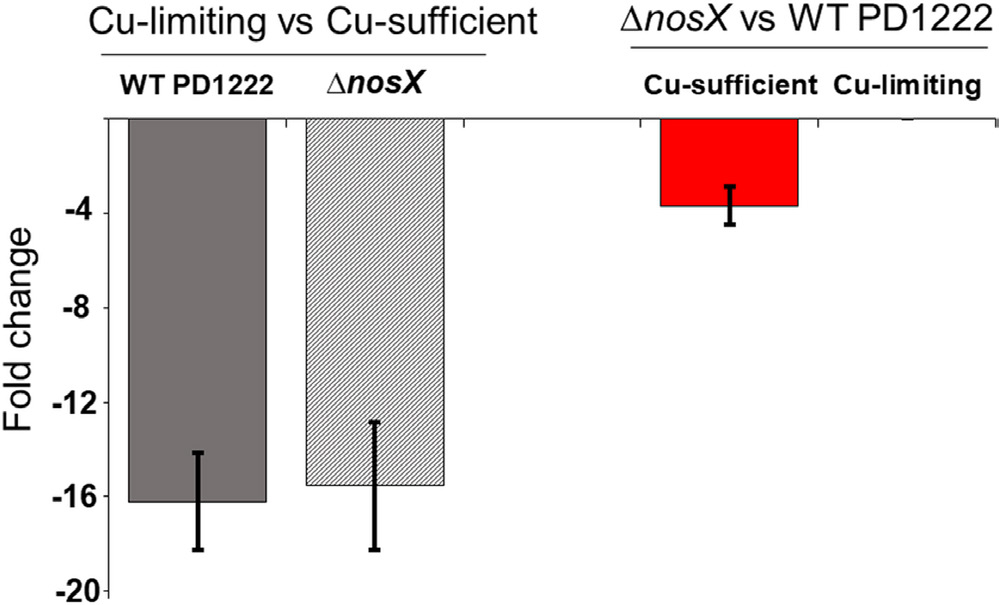
*nosZ* relative expression determined by qRT-PCR. Left side shows *nosZ* expression change under Cu-limited versus Cu-sufficient conditions in the WT PD1222 and Δ*nosX* mutant strains. Right side shows *nosZ* expression change in Δ*nosX* mutant versus WT PD1222 strains under Cu-limiting and Cu-sufficient growth conditions.

To investigate this, qRT-PCR experiments were performed to determine the differential expression of *nosZ* in the *nosX* mutant compared to wild-type cells. Under Cu-sufficient conditions, a twofold decrease (1.9±0.2) in expression of *nosZ* was measured in Δ*nosX* compared to wild-type cells (Fig. 5). This likely contributes to the observed increased incorporation of Cu into step-tagged N_2_OR isolated from the Δ*nosX* mutant compared to that from wild-type cells. However, no significant difference in expression of *nosZ* was detected between Δ*nosX* and wild-type grown under Cu-limiting conditions. In both cases, the *nosZ* expression in Cu-limiting conditions was ~15-fold lower than that under Cu-sufficient conditions (Fig. 5), consistent with previous report on the effect of Cu on *nosZ* expression in wild-type cells [17]. Thus, effects on *nosZ* expression do not account for the very low incorporation of Cu into strep-tagged N_2_OR in wild-type cells compared to in the Δ*nosX* mutant.

## Discussion

The *nosX* gene is conserved across the NGC of α- and β-proteobacteria, but not among γ- or clade-II members of N_2_O-reducing bacteria. Here, we have demonstrated a Nos^-^ phenotype for a *nosX* deletion mutant in *P. denitrificans* (PD2502), which was complemented *in trans* using a functional *nosX* plasmid-borne gene copy under taurine inducible control. NosX is a member of the AbpE protein family, which bind flavin adenine dinucleotide [33, 34]. Some AbpE proteins are flavinyl transferases, functioning in the post-translational maturation of another flavin-requiring protein. For example, *Vibrio cholera* ApbE transfers a flavin mononucleotide (FMN) to a threonine residue in NqrC [35]. In *P. denitrificans* there are three *abpE* homologues: *nosX,* encoded by *pden_4214*, *nirX* (*pden_2485*) and *pden_3291*. NosX and NirX are exported to the periplasm via the Tat pathway while Pden_3291 is predicted to be cytoplasmic.

An earlier study of an antibiotic cassette insertion mutation in the *P. dentrificans nosX* gene reported no effect on N_2_OR activity [22]. This led to the proposal that the *nirX* gene in *P. denitrificans* is a functional homologue of *nosX*, such that mutation of both genes are required in order to observe a Nos^-^ phenotype. This previous conclusion is clearly at odds with the data presented here. One possibly important observation is that the previous mutagenesis study did not involve full *nosX* deletion. Conserved residues within the putative FAD binding pocket in NosX are now known, including Ser68, Tyr70, Thr174 and Gly256, based on sequence similarities with the *Se*ApbE (Fig. S1) [34]. If these residues are important for NosX function, then the previous mutation strategy for *P. dentrificans nosX,* in which a kanamycin insertion was made 469 bp into the gene, would not have disrupted the conserved Ser68 and Tyr70 residues. The resulting truncated NosX may have retained some function, which would account for why a clear phenotype was not observed in the single *nosX* insertional mutant. We note that the requirement for *nosX* in N_2_O reduction has also been demonstrated in *Sinorhizobium meliloti *. In that case, a Tn*5-*mediated mutation 31 nucleotides into the total 966 nucleotide sequence downstream of *nosDFYL*, a region now recognized as *nosX*, abolished N_2_OR activity [36].

In the earlier report of a double *nosXnirX* mutant of *P. denitrificans *, it was reported that the N_2_OR present in unfractionated periplasm from this mutant was deficient in the Cu_A_ centre, leading to the conclusion that NosX and NirX play a role in assembly of this cofactor [22]. However, subsequent studies of anaerobically purified N_2_OR from the double *nirXnosX* mutant and a single *nirX* mutant indicated that the absence of NosX resulted in N_2_OR with both Cu cofactors assembled, but with Cu_Z_ exhibiting a spectroscopically distinct from, termed pink Cu_Z_*, that is normally only observed upon reaction with O_2_ [37]. This Cu_Z_ form is not catalytically active, but is proposed to represent a catalytically relevant intermediate oxidation state of the Cu_Z_ centre ([4CuS]^3+^), which binds N_2_O and proceeds through a state denoted as Cu_Z_^0^ [38].

Here, to determine the effect of the absence of *nosX*/NosX alone on N_2_OR, we utilized a previously reported plasmid-encoded Strep-tagged N_2_OR that can be readily isolated from different background strains and characterized in terms of its Cu cofactor content and spectroscopic properties. These experiments demonstrated unequivocally that the assembly of the Cu_A_ and Cu_Z_ centres was unaffected in the absence of *nosX*. Thus, the phenotype exhibited by the mutant does not arise because of a deficiency in the insertion of Cu into N_2_OR. We note that the spectroscopic properties of N_2_OR from the Δ*nosX* mutant strain are the same as those of the Cu_Z_* centre from purified from the *nosXnirX* mutant. This may suggest the Cu_Z_ centre was purified in a catalytically inactive redox state. However, the pink form reported in this work was generated by aerobic purification, with *nirX* remaining in the genome and under conditions where we expect to observe the Cu_Z_ centre is this pink Cu_Z_* form, as demonstrated by the control experiments with N_2_OR isolated from the wild-type strain.

ApbE from the N_2_O-reducing bacterium *P. stutzeri* is a monomeric FAD-binding protein [23]. In the absence of *nosX* in the NGC of *P. stutzeri,* AbpE functions as a flavin donor, catalysing the covalent flavinylation of a threonine residue of NosR [23]. Importantly, the post-translationally modified, FMN-bound NosR is proposed to be the electron donor to N_2_OR, such that in the absence of NosR N_2_OR is not functional. Our data indicate that N_2_OR Cu cofactor maturation is unaffected by the loss of NosX, and we conclude that in *P. denitrificans* it most likely functions as the main system-specific maturation factor for NosR, and thus as an indirect activator of N_2_OR. If this is the case, then a Nos^-^ phenotype would be expected for a Δ*nosR* strain. This was recently demonstrated: a *P. denitrificans* Δ*nosR* strain exhibited a vastly decreased capacity to reduce N_2_O, irrespective of the levels of Cu in the cell [17]. However, we note that the Δ*nosR* strain did retain some ability to reduce N_2_O, whereas the *nosX* mutant investigated here did not, and so the *nosX* phenotype is actually more dramatic than the *nosR* phenotype. Why this is the case is not clear. One possibility is that NosX does not only mature NosR, such that in the absence of NosX, there is a further effect on NosZ activity. Alternatively, having a non-flavinylated NosR present might somehow inhibit NosZ more severely than having no NosR present at all. We also note that the previously reported transcription data revealed the loss of Cu-responsive transcription of *nosZ* in the *nosR* deletion strain [17], suggesting that NosR itself may be multifunctional, or that its absence leads to pleiotropic effects, some of which may be indirect. Clearly, further studies are needed to investigate directly the role of NosX in NosR maturation, and more generally other possible roles of NosX and the function(s) of NosR.

An intriguing observation reported here is the lower levels of Cu cofactor incorporation observed under Cu-limited conditions for the Strep-tagged N_2_OR from wild-type cells compared to that from the *nosZ* and *nosX* mutants. One possibility that we examined was that *nosX*/NosX is involved in the regulation of *nosZ*, such that in the absence of *nosX*/NosX, lower amounts of chromosomally encoded N_2_OR were present, perhaps leading to less competition for copper and higher incorporation of Cu into the plasmid-encoded Strep-tagged form. While the absence of *nosX* did result in a twofold reduction of *nosZ* expression under Cu-sufficient conditions, no significant difference between the wild-type and *nosX* mutant strains was detected under Cu-limited conditions where the incorporation of Cu was most pronounced. The very low expression of the chromosomal *nosZ* gene under Cu-limited conditions suggests that a simple competition between chromosomal- and plasmid-encoded N_2_OR enzymes for Cu is unlikely. A further possibility is that the presence of the Strep-tag required for rapid recovery and biochemical analysis of NosZ results in modest perturbation of Cu cofactor assembly factor interactions such that the wild-type enzyme is a preferred substrate, an effect that only becomes apparent under very low Cu conditions. Clearly, further studies are needed to explore this possibility.

In summary, the data presented here show that *nosX* is essential for whole-cell N_2_O reduction in the α-proteobacterium *P. denitrificans *, and that the *nosX* and *nirX* gene products are not functionally redundant under our experimental conditions. The function of NosX is not associated with the assembly of the Cu cofactors of N_2_OR. Instead, based on homology between NosX and ApbE proteins, and the recent demonstration of an essential role for an ApbE family flavin transferase in the maturation of NosR in *P. stutzeri *, it is likely that NosX is involved in indirectly maintaining the reaction cycle of N_2_OR through the flavinylation of another accessory protein, NosR.

## Supplementary Data

Supplementary material 1
