# Supplementary material for: nosX is essential for whole-cell N2O reduction in Paracoccus denitrificans but not for assembly of copper centres of nitrous oxide reductase
Source: Microbiology (Reading). 2020 Sep 4;166(10):909–17. doi: 10.1099/mic.0.000955 (PMC7660919; doi:10.1099/mic.0.000955)
Supplement: Supplementary material 1 [file mic-166-909-s001.pdf]

Supporting data

nosX is essential for whole cell N_2O reduction in *Paracoccus denitrificans* but not for assembly of copper centres of nitrous oxide reductase.

Sophie P. Bennett, Maria J. Torres, Manuel J. Soriano-Laguna, David J. Richardson, Andrew J. Gates, Nick E. Le Brun

Supporting Tables

Table S1. Strains and plasmids used in this study.

Name	Relevant Characteristics	Source
Bacterial strains		
P. denitrificans PD1222	Wild-type strain, $spec^R$	[1]
P. denitrificans PD2303	Unmarked Δ*nosZ* deletion mutant, $spec^R$	[2]
E. coli JM101	Used as host for pK18*mobsacB*-based plasmids	[3]
P. denitrificans PD2502	Unmarked Δ*nosX* deletion mutant, $spec^R$	This work
E. coli DH5α	Used as host for plasmid modification/propagation	[4]
Plasmids		
pRK2013	Used as mobilizing plasmid in triparental crosses, kan^R	[5]
pK18*mobsacB*	Allelic exchange suicide plasmid, sucrose-sensitive, mob^+, kan^R	[6]
pSPBN4	*pk18mobsacB*-derivative, construct for *nosX* deletion, kan^R	[7]
pSPBN5	Complementation plasmid for *nosX* from *P. denitrificans* (Pden_4214 sequence cloned into pLMB511 as a *Nde*-I – *Eco*RI fragment) gen^R	This work
pMSL002	pLMB511 expression construct for strep-II tagged *P. denitrificans* NosZ (Pden_4219 sequence), gen^R	[7]

Supporting Figures

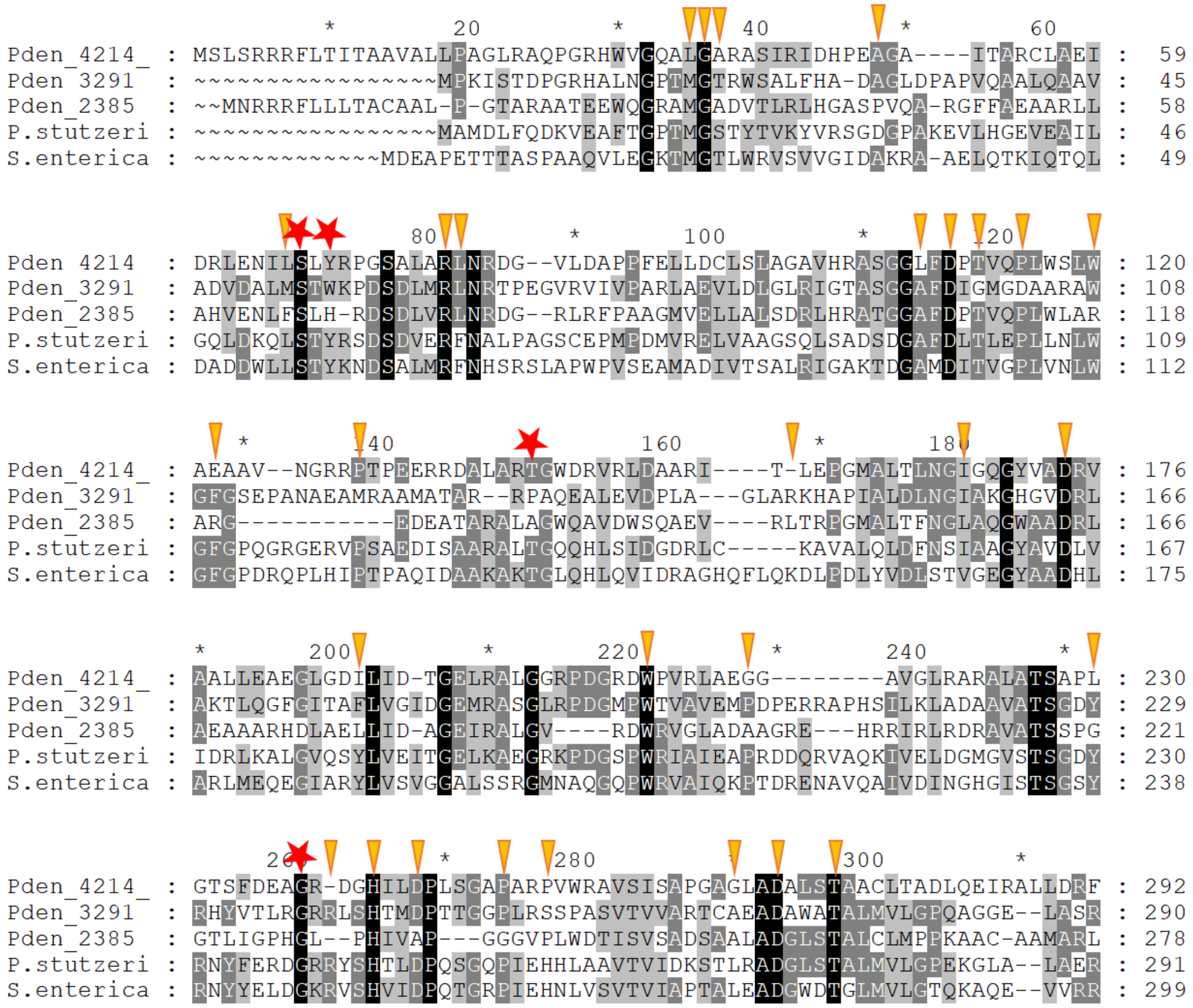

Fig. S1. Amino acid residue sequence alignment of AbpE-family proteins. Pden_4214 (NosX), Pden_2485 (NirX) and Pden_3291 from *P. denitrificans* were aligned with *P. stutzeri* and *S. enterica* ApbE-family proteins. 100% and 60% conserved residues are indicated in black and grey, respectively. Highly conserved residues across other AbpE proteins from α-proteobacteria, determined by a Blast search, are revealed by yellow triangles. Ser68, Tyr70, Thr174 and Gly256 are also conserved in *S. enterica* AbpE, in which FAD-binding involves these residues (red stars).

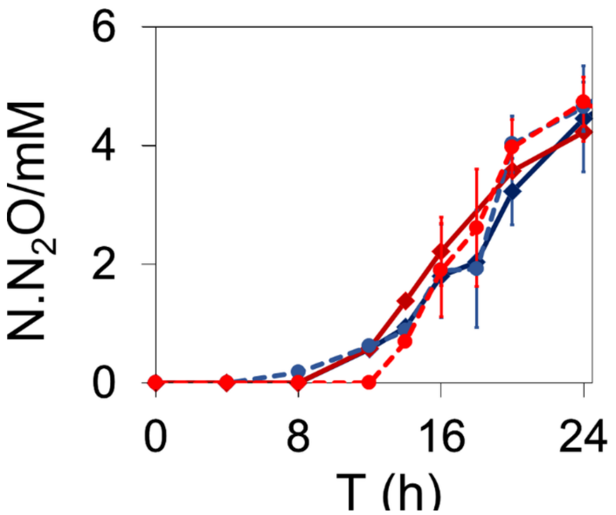

Fig. S2. Overlaid plot of N_2O production due to *P. denitrificans* Δ*nosZ and* Δ*nosX* mutant strains. N_2O emissions as $N.N_2O$ (millimolar N in the form of N_2O) for grown in anaerobic batch culture in Cu-sufficient media (red symbols) and Cu-limited media (blue symbols). Data for Δ*nosZ* mutant are in dark shade diamonds with solid lines, while data for Δ*nosX* deletion mutant are in lighter shade circles with dashed lines. Cultures were grown in triplicate and bars represent SE. The data are replotted from Fig. 2b and c of the main paper.

Supporting references

1. **de Vries GE, Harms N, Hoogendijk J, Stouthamer AH**. Isolation and characterization of *Paracoccus denitrificans* mutants with increased conjugation frequencies and pleiotropic loss of a (nGATCn) DNA-modifying property. *Arch Microbiol* 1989;152:52-57.
2. **Sullivan MJ, Gates AJ, Appia-Ayme C, Rowley G, Richardson DJ**. Copper control of bacterial nitrous oxide emission and its imact on vitamin B_{12}-dependent metabolism. *Proc Natl Acad Sci USA* 2013;110:19926-19931.
3. **Messing J**. A multipurpose cloning system based on the single stranded DNA bacteriophage M13. *Recomb DNA Tech Bull* 1979;2:43-48.
4. **Hanahan D**. Studies on transformation of Escherichia coli with plasmids. *J Mol Biol* 1983;166:557-580.
5. **Figurski DH, Helinski DR**. Replication of an origin-containing derivative of plasmid RK2 dependent on a plasmid function provided in trans. *Proc Natl Acad Sci USA* 1979;76:1648-1652.
6. **Schafer A, Tauch A, Jager W, Kalinowski J, Thierbach G et al.** Small mobilizable multi-purpose cloning vectors derived from the *Escherichia coli* plasmids pK18 and pK19: selection of defined deletions in the chromosome of *Corynebacterium glutamicum*. *Gene* 1994;145:69-73.
7. **Bennett SP, Soriano-Laguna MJ, Bradley Justin M, Svistunenko DA, Richardson DJ et al.** NosL is a dedicated copper chaperone for assembly of the CuZ center of nitrous oxide reductase. *Chem Sci*, 2019;10:4985-4993.
